# Machine Learning for Multi-Target Drug Discovery: Challenges and Opportunities in Systems Pharmacology

**DOI:** 10.3390/pharmaceutics17091186

**Published:** 2025-09-12

**Authors:** Xueyuan Bi, Yangyang Wang, Jihan Wang, Cuicui Liu

**Affiliations:** 1 Department of Pharmacy, Honghui Hospital, Xi’an Jiaotong University, Xi’an 710054, China; 2 School of Physics and Electronic Information, Yan’an University, Yan’an 716000, China; 3 Yan’an Medical College of Yan’an University, Yan’an 716000, China; 4 Department of Science and Education, Honghui Hospital, Xi’an Jiaotong University, Xi’an 710054, China

**Keywords:** machine learning, multi-target drug discovery, systems pharmacology, deep learning, graph neural networks, drug repurposing, network pharmacology, pharmacokinetics

## Abstract

Multi-target drug discovery has become an essential strategy for treating complex diseases involving multiple molecular pathways. Traditional single-target approaches often fall short in addressing the multifactorial nature of conditions such as cancer and neurodegenerative disorders. With the rise in large-scale biological data and algorithmic advances, machine learning (ML) has emerged as a powerful tool to accelerate and optimize multi-target drug development. This review presents a comprehensive overview of ML techniques, including advanced deep learning (DL) approaches like attention-based models, and highlights their application in multi-target prediction, from traditional supervised learning to modern graph-based and multi-task learning frameworks. We highlight real-world applications in oncology, central nervous system disorders, and drug repurposing, showcasing the translational potential of ML in systems pharmacology. Major challenges are discussed, such as data sparsity, lack of interpretability, limited generalizability, and integration into experimental workflows. We also address ethical and regulatory considerations surrounding model transparency, fairness, and reproducibility. Looking forward, we explore promising directions such as generative modeling, federated learning, and patient-specific therapy design. Together, these advances point toward a future of precision polypharmacology driven by biologically informed and interpretable ML models. This review aims to provide researchers and practitioners with a roadmap for leveraging ML in the development of safer and more effective multi-target therapeutics.

## 1. Introduction

In recent years, the paradigm of drug discovery has been shifting from the traditional “one drug, one target” approach toward a more holistic and systems-level strategy—multi-target drug discovery [1,2]. This transformation is driven by the growing recognition that complex diseases such as cancer, neurodegenerative disorders, and metabolic syndromes often involve dysregulation of multiple genes, proteins, and pathways [3]. As a result, modulating a single molecular target may offer limited therapeutic benefit, whereas a well-designed multi-target intervention can improve efficacy, reduce resistance, and enhance safety profiles. This concept aligns with the principles of systems pharmacology, which integrates network biology, pharmacokinetics/pharmacodynamics (PK/PD), and computational modeling to understand drug action at the systems level [4].

Multi-target drugs are strategically designed to interact with a pre-defined set of molecular targets to achieve a synergistic therapeutic effect for complex diseases. This approach, known as rational polypharmacology, is a deliberate strategy to address the multifactorial nature of diseases [5,6]. In contrast, promiscuous drugs are compounds that exhibit a lack of specificity, binding to a wide range of unintended targets, which often leads to off-target effects and toxicity. While both types of drugs interact with multiple targets, the key distinction lies in the intentionality and specificity of their design. A multi-target drug’s target spectrum is carefully selected to contribute to the desired therapeutic outcome, whereas a promiscuous drug’s binding is often broad and unselective, potentially including many irrelevant or undesirable targets. Therefore, while a multi-target drug is by nature a ‘promiscuous’ binder, the term ‘promiscuous’ is often used pejoratively to denote a lack of specificity, whereas ‘multi-target’ implies a deliberate and beneficial polypharmacology [7].

However, identifying effective multi-target drug candidates remains a major challenge. The combinatorial explosion of potential drug-target interactions (DTI), the complexity of biological networks, and the limitations of high-throughput experimental screening demand scalable, accurate, and intelligent computational solutions [8]. In this context, machine learning (ML) has emerged as a powerful toolkit for modeling complex, nonlinear relationships inherent in biological systems [9,10]. By learning from diverse data sources—such as molecular structures, omics profiles, protein interactions, and clinical outcomes—ML algorithms can prioritize promising drug-target pairs, predict off-target effects, and even propose novel compounds with desirable polypharmacological profiles [11,12,13].

The integration of ML into multi-target drug discovery has opened new opportunities for understanding and optimizing complex therapeutic strategies, but it has also introduced significant challenges in data representation, model generalizability, and clinical applicability [14,15,16]. Classical ML models, such as support vector machines (SVMs), random forests (RFs), and logistic regression, have long demonstrated utility in tasks like predicting DTI, adverse effects, and pharmacokinetic profiles [17]. These models benefit from interpretability and robustness when trained on curated datasets. However, the growing volume and complexity of biomedical data have spurred the adoption of more sophisticated deep learning (DL) architectures [18,19]. In particular, graph neural networks (GNNs) excel at learning from molecular graphs and biological networks, while transformer-based models are increasingly leveraged to capture sequential, contextual, and multimodal biological information [18,20]. These approaches allow for the integration of chemical structure, target profiles, gene expression, and clinical phenotypes into unified predictive frameworks. Furthermore, the incorporation of systems pharmacology principles enables ML models to go beyond molecule-level predictions by considering the effects of drugs across pathways, tissues, and disease networks, facilitating a more holistic view of therapeutic efficacy and safety [21].

**Figure 1:**
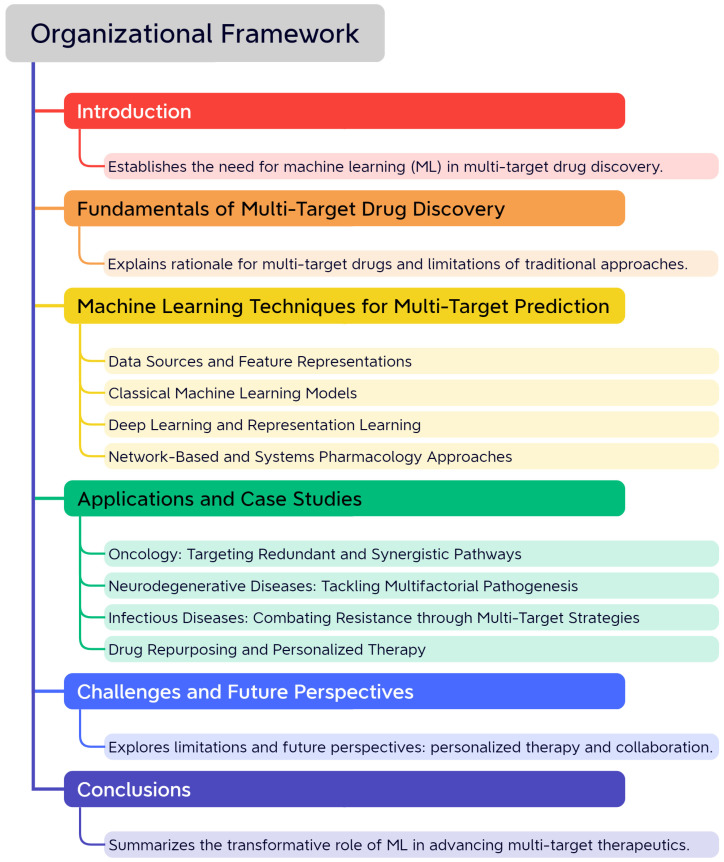
Organizational Framework of the Review. This mind map provides a comprehensive overview of the article’s structure, illustrating the logical flow from the introduction of multi-target drug discovery to the fundamental concepts, advanced machine learning techniques, real-world applications, and concluding with challenges and future perspectives.

This review provides a comprehensive overview of how ML methods are applied to multi-target drug discovery, with an emphasis on their integration into systems pharmacology. We begin by outlining the biological rationale, then explore various ML techniques for feature representation, model construction, and network-based analysis. Key applications and case studies will be discussed to highlight their real-world implications. Finally, we address current challenges and propose future directions to guide research at the intersection of AI and multi-target pharmacology. The organizational framework is presented in Figure 1.

## 2. Fundamentals of Multi-Target Drug Discovery

**Figure 2:**
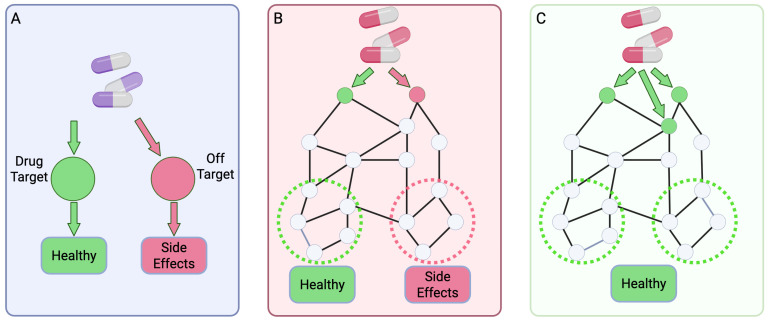
Comparison of Therapeutic Strategies. (**A**) The classic “one-drug, one-target” model, where a single drug is designed to modulate a specific target to achieve a therapeutic effect. Off-target binding can lead to unintended side effects. (**B**) A systems biology view, which considers how a drug’s interaction with a single target can propagate through a complex biological network, leading to both desired therapeutic effects and potential side effects. (**C**) The systems pharmacology approach, where a single drug is designed to act on multiple targets or a network of targets to produce a synergistic therapeutic effect and restore network stability, thus minimizing side effects.

Traditional drug discovery often follows a ‘one-drug, one-target’ paradigm, but this approach has limitations when addressing complex diseases. The evolution from this classical view to a systems pharmacology perspective is illustrated in Figure 2, which highlights the shift from single-target to multi-target and network-based therapeutic strategies.

The traditional paradigm of drug discovery has long focused on developing selective agents that modulate a single target associated with a specific disease [22]. While this approach has achieved success in certain therapeutic areas, it often falls short when applied to complex, multifactorial diseases such as cancer, neurodegenerative disorders, cardiovascular disease, and autoimmune conditions [23]. These diseases typically involve intricate biological networks with multiple dysregulated genes, signaling pathways, and feedback loops [24]. Consequently, interventions targeting a single node in such networks may lead to suboptimal efficacy, rapid resistance development, or unintended compensatory mechanisms. Multi-target drug discovery, or polypharmacology, offers a promising alternative by aiming to simultaneously modulate multiple targets involved in disease progression [25]. This strategy can produce synergistic therapeutic effects, enhance efficacy, and improve safety by reducing the doses required for each target [26]. In oncology, for instance, multi-kinase inhibitors have demonstrated improved clinical outcomes by blocking redundant signaling pathways that contribute to tumor survival [27]. In neurodegenerative diseases, addressing both amyloid accumulation and neuroinflammation through dual-target mechanisms holds greater promise than targeting either pathway alone [28,29]. Moreover, the concept of “network pharmacology” emphasizes that diseases are not the result of a single gene malfunction but rather the perturbation of interconnected networks [30]. From this perspective, therapeutic strategies should aim to restore network stability rather than simply block an individual target [31]. This system-level understanding forms the biological rationale for designing drugs that act on multiple molecular entities in a coordinated manner.

Despite the appeal of multi-target drug discovery, conventional experimental methods face significant limitations [23]. A major challenge lies in the combinatorial explosion of possible target sets and compound-target interactions [26]. With thousands of potential targets and millions of chemical compounds, the search space for discovering effective multi-target combinations becomes intractable using brute-force experimental techniques alone. Furthermore, traditional computational approaches—such as molecular docking or ligand-based virtual screening—often rely on predefined assumptions and simplified representations of molecular interactions [32,33]. Given these limitations, there is a pressing need for more sophisticated, data-driven approaches that can navigate the high-dimensional, nonlinear space of drug-target-disease interactions.

## 3. Machine Learning Techniques for Multi-Target Prediction

**Figure 3:**
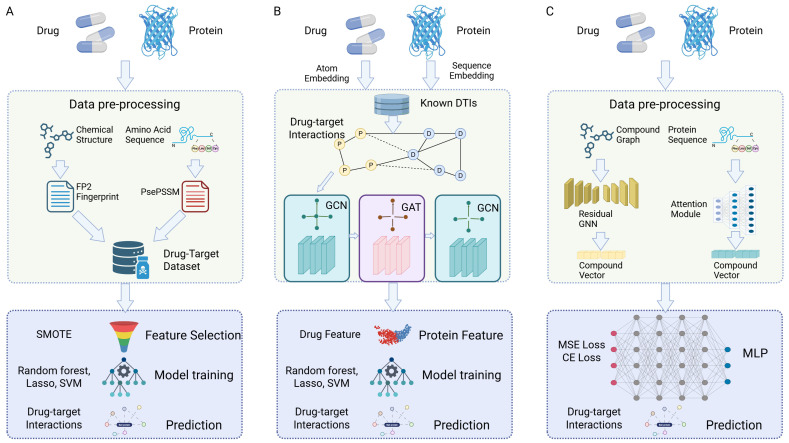
Representative Machine Learning Pipelines for Drug-Target Prediction. The figure illustrates three different computational workflows. (**A**) A classical ML pipeline that relies on feature engineering, where chemical structures and protein sequences are converted into handcrafted features (e.g., FP2 fingerprints, PsePSSM) before being used to train traditional models like RF, Lasso, or SVM for prediction. (**B**) A DL pipeline that utilizes GNNs for representation learning, where drugs and proteins are represented as nodes in a graph and GNNs, including variants like graph convolutional networks (GCNs) and graph attention networks (GATs), automatically learn features to predict DTI. (**C**) A modern DL pipeline that integrates advanced architectures, such as a residual GNN for compound representation and an attention module for protein sequence embedding, which are then fused and processed by a multi-layer perceptron (MLP) for final prediction. This approach leverages the strengths of both graph-based and attention-based models to capture complex relationships.

The complex and nonlinear nature of multi-target drug discovery requires computational methods that can efficiently model interactions across diverse chemical and biological spaces. ML has become a powerful approach to address these challenges, offering the flexibility to integrate heterogeneous data, learn hidden patterns, and make predictions at scale [34]. Figure 3 provides a visual overview of several representative pipelines, ranging from classical feature-based methods to modern DL architectures that leverage graph-based and attention mechanisms for more sophisticated predictions. This section outlines the key components of ML systems used for multi-target prediction, including data representations, classical and DL models, and network-based systems pharmacology frameworks.

### 3.1. Data Sources and Feature Representations

Effective ML for multi-target drug discovery relies heavily on rich, well-structured data representations derived from diverse biological and chemical domains [35]. Drug molecules can be encoded using a variety of representations, including molecular fingerprints (e.g., ECFP), SMILES strings, handcrafted molecular descriptors, and graph-based encodings that preserve structural topology [36,37]. On the other hand, targets—typically proteins—can be represented by their amino acid sequences, structural conformations, or contextual positions in protein–protein interaction (PPI) networks [38,39,40]. Modern embedding techniques such as those from pre-trained protein language models (e.g., ESM, ProtBERT) [41] and graph-based node embedding algorithms (e.g., DeepWalk, node2vec) [42] enable transformation of these entities into vectorized forms suitable for ML. Interaction data such as drug-target binding affinities or multi-label activity profiles are often collected from databases like DrugBank, ChEMBL, BindingDB, and STITCH [43]. A more comprehensive list of these and other key data sources is provided in Table 1. A central challenge is integrating such heterogeneous features into a unified learning framework [44], often addressed via feature fusion, co-embedding strategies, or representation learning.

### 3.2. Classical Machine Learning Models

Classical ML algorithms have laid the groundwork for multi-target drug discovery by offering scalable and interpretable tools for prediction. SVMs and RFs are widely adopted due to their strong performance in high-dimensional settings and robustness to overfitting [46,47]. These models have been used for both binary classification and regression tasks in DTI prediction. Other models such as k-nearest neighbors (k-NN) and naïve Bayes (NB) classifiers offer simplicity and speed, although they may lack the capacity to model complex relationships [48]. Multi-label classification strategies, including binary relevance, classifier chains, and label powerset transformations, have been employed to model drugs acting on multiple targets simultaneously [49]. However, classical models generally require extensive feature engineering and may not capture the higher-order dependencies found in biological systems, limiting their performance in highly nonlinear and interconnected pharmacological landscapes.

### 3.3. Deep Learning and Representation Learning

DL approaches have significantly advanced the capabilities of multi-target drug prediction by enabling automatic feature extraction and modeling of complex nonlinearities [50,51]. Multi-task learning (MTL) frameworks are particularly suited for multi-target settings, as they learn shared representations across related prediction tasks [52]. GNNs [53], including variants like graph convolutional networks (GCNs), graph attention networks (GATs), and GraphSAGE [54,55], have proven effective at modeling molecular graphs and predicting interactions by capturing rich spatial and relational information. A key architectural enhancement, residual GNNs, addresses the challenge of vanishing gradients in deep networks. By adding “skip connections” that allow information to bypass one or more layers, residual GNNs enable the training of much deeper models, facilitating the capture of complex, long-range dependencies within a molecule’s topological structure without losing crucial information from initial layers. This makes them particularly suitable for representing large and intricate chemical compounds [56]. Transformer-based models [57,58], originally developed for language modeling, are now applied to SMILES strings and protein sequences, with models like ChemBERTa [59], SMILES-BERT [60], and ProtTrans [61] demonstrating strong generalization in limited data scenarios. The core innovation of the Transformer is the self-attention mechanism. Unlike recurrent neural networks (RNNs) that process sequences sequentially, the attention mechanism allows the model to weigh the importance of all amino acids in a protein sequence simultaneously, regardless of their position. This parallel processing not only significantly improves computational efficiency but, more critically, allows the model to directly capture long-range dependencies and interactions between distant amino acids in a protein’s primary structure, which are often vital for its function and 3D folding. This capability makes Transformers a superior alternative for protein sequence representation compared to traditional sequential models [62]. Additionally, more refined multi-head attention-based models have been developed specifically for drug repurposing. These models leverage large-scale biological datasets to predict drug–disease relationships and demonstrate robust generalization capabilities, with some of their predictions indirectly confirmed by clinical trials [63]. Furthermore, unsupervised techniques such as autoencoder (AE), variational autoencoder (VAE) [64], and contrastive learning approaches allow the construction of latent representations even when labeled data are sparse [65]. Despite their predictive power, deep models often suffer from interpretability issues [66], raising concerns in clinical and regulatory settings.

The advent of generative artificial intelligence (AI) has revolutionized the paradigm of multi-target drug discovery by shifting from a traditional ‘screen-and-select’ approach to a ‘design-and-generate’ one. These models, trained on vast datasets of chemical structures and their associated biological activities, can learn the underlying chemical and pharmacological rules to create entirely novel molecules from scratch, a process known as de novo design [14,67]. A key advantage of generative AI in this context is its ability to explore the immense chemical space more effectively than traditional methods. Models such as VAEs, generative adversarial networks (GANs) [68], and diffusion models [69] are used to produce molecular structures with specific, pre-defined properties, including the desired multi-target profiles. These models are particularly suited for polypharmacology because they can be optimized to generate compounds that simultaneously satisfy multiple objectives, such as binding affinity to several targets, drug-likeness, and synthetic accessibility [70]. For instance, recent studies have demonstrated the use of generative models to design multi-target ligands for complex diseases, showcasing their potential to accelerate the development of more effective and safer therapeutics [71,72].

In summary, each of the discussed approaches offers distinct trade-offs in multi-target drug discovery. Classical ML models (Section 3.2) excel in tasks with limited, well-defined data, offering high interpretability and lower computational costs [73]. However, their reliance on manual feature engineering and limited capacity to model complex, hierarchical relationships hinder their scalability for large, high-dimensional datasets. In contrast, DL methods (Section 3.3), particularly GNNs and Transformers, demonstrate superior performance and scalability by automatically learning complex patterns from vast, heterogeneous data [74]. While DL excels in predictive power, it often suffers from poor interpretability, a critical limitation in a field requiring high-stakes, explainable decisions. The emerging systems pharmacology approaches and hybrid models (Section 3.4) bridge this gap by integrating diverse data sources and network contexts. They offer a promising path to not only improve predictive accuracy but also enhance the biological relevance and interpretability of results, making them highly suitable for complex, systems-level therapeutic tasks like multi-target drug repurposing and de novo design [5].

### 3.4. Network-Based and Systems Pharmacology Approaches

ML in systems pharmacology often leverages the structure of biological networks to contextualize DTI [75,76]. Heterogeneous networks, which represent drugs, proteins, diseases, and pathways as interconnected nodes, offer a rich substrate for prediction via link prediction or network completion algorithms [77]. Embedding methods such as node2vec [78], metapath2vec [79], and graph AE allow transformation of these complex networks into low-dimensional feature spaces while preserving topological and semantic relationships. In addition, network propagation [80] and diffusion algorithms [81] simulate how drug perturbations influence biological systems, identifying synergistic multi-target profiles and compensatory pathways. Knowledge graphs further extend these capabilities by encoding semantic relations among biomedical entities [82]; when combined with embedding methods like TransE or GNN-based link predictors [83], they support reasoning over complex associations and contribute to explainable predictions [78]. These systems-level approaches provide the necessary context for understanding polypharmacology beyond molecular interactions and support network-informed therapeutic design. These methods can identify not just potential drug candidates but also the optimal network nodes to modulate for desired therapeutic outcomes.

A key trend is to integrate factors beyond direct drug-target binding, such as pharmacokinetics and the influence of the gut microbiota (GM). A recent study using an animal model showed that the GM can significantly alter the pharmacokinetics of drugs, thereby affecting their efficacy and potential for drug interactions [84]. This highlights the importance of a systems-level approach that considers not only molecular interactions but also physiological and microbial factors to provide a more complete picture of drug action.

The ability of knowledge graphs to encode vast, heterogeneous biomedical data makes them particularly powerful for novel target discovery and drug repurposing, which are essential facets of multi-target therapy. Recent advancements have led to the development of sophisticated graph foundation models that perform zero-shot drug repurposing, identifying therapeutic candidates for diseases with limited or no existing treatments. A prime example is TxGNN, a graph neural network model trained on a comprehensive medical knowledge graph [85]. TxGNN uses a metric learning module to rank potential drug indications and contraindications, showcasing a significant improvement in prediction accuracy over conventional methods. A key feature of such models is their interpretability, facilitated by modules like TxGNN’s Explainer. This component provides transparent, multi-hop knowledge paths that allow human experts to investigate and validate the model’s predictions, thereby building trust and bridging the gap between computational results and clinical decision-making. These innovations highlight the potential of knowledge graphs not only for predicting known DTI but also for discovering entirely new therapeutic connections within the complex landscape of diseases. Beyond the molecular level, the scope of multi-target therapy extends to encompass the systemic, multi-organ nature of chronic diseases. This holistic perspective is crucial because many complex conditions, such as metabolic or neurodegenerative disorders, involve an intricate web of malfunctioning organs and disrupted inter-organ communication. A recent concept, the locked-state model (LoSM), illustrates how these pathological states can become ‘locked’ due to body-wide, memory-like properties resulting from inter-organ crosstalk [86]. Deciphering these complex, multi-organ locked states present a significant opportunity for systems pharmacology. Applying ML and systems biology at this multi-scale level enables the identification of therapeutic strategies that can dismantle pathological states by targeting key nodes within these complex biological systems. This approach not only facilitates the discovery of optimal multi-target profiles but also helps in designing interventions that can modulate an entire system rather than just a single pathway. These advancements highlight a new frontier in multi-target therapy, where the goal is to develop treatments that restore healthy inter-organ communication and unlock chronic pathological states.

## 4. Applications and Case Studies

**Figure 4:**
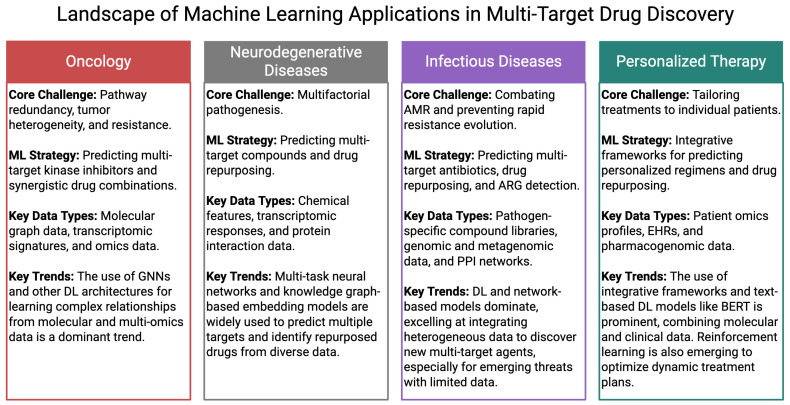
A Comparative Overview of Machine Learning in Multi-Target Drug Discovery. This figure summarizes the key trends and strategies of ML applications across four critical therapeutic areas.

ML-based strategies for multi-target drug discovery have transitioned from theoretical frameworks to practical applications across a wide spectrum of therapeutic areas. These applications demonstrate the feasibility of ML techniques and highlight their capacity to uncover novel DTI, optimize combination therapies, and accelerate drug repurposing. This section presents representative case studies that illustrate how ML is being applied to real-world drug discovery challenges in oncology, neurodegenerative diseases, infectious diseases, and personalized therapy. As shown in Figure 4, these diverse applications reveal a common theme: ML models are evolving to integrate different data types and address the unique, multifaceted challenges of each therapeutic area, paving the way for a new era of precision polypharmacology.

### 4.1. Oncology: Targeting Redundant and Synergistic Pathways

As highlighted in Figure 4, cancer presents a primary area for ML-guided multi-target drug discovery due to its intrinsic biological complexity, including pathway redundancy, tumor heterogeneity, and adaptive resistance mechanisms [87,88]. The use of multi-target therapeutics in oncology is crucial for overcoming resistance and achieving synergistic effects. Table 2 provides a summary of representative studies that utilize ML for drug combination and synergy prediction in cancer treatment. ML models have been applied to identify kinase inhibitors capable of targeting multiple oncogenic pathways simultaneously. For instance, DL models integrating molecular graph data and transcriptomic signatures have been used to predict dual or triple kinase inhibitors with improved efficacy across different cancer cell lines [89,90]. In another example, a graph-based ML framework was used to identify multitarget compounds capable of co-inhibiting PI3K, mTOR, and EGFR—three pathways frequently co-activated in lung and breast cancers [91,92,93]. These predictions were validated with in vitro and xenograft studies, underscoring the translational potential of ML-guided approaches. Additionally, ML-assisted drug combination prediction has emerged as a complementary strategy, where models forecast synergistic drug pairs based on cell line–specific omics data and pathway activity scores, enabling more effective multi-target treatment regimens [94].

### 4.2. Neurodegenerative Diseases: Tackling Multifactorial Pathogenesis

Neurodegenerative diseases such as Alzheimer’s and Parkinson’s disease (AD and PD) involve multiple pathogenic processes, including protein aggregation, mitochondrial dysfunction, oxidative stress, and neuroinflammation [118]. Given the multifactorial pathogenesis of neurodegenerative disorders, multi-target strategies are particularly well-suited for their treatment. A summary of key studies that have employed ML for multi-target drug discovery in this area is provided in Table 3. Conventional single-target strategies have failed to deliver effective disease-modifying therapies, prompting a shift toward systems-level approaches. ML models have been trained on chemical features, transcriptomic responses, and protein interaction data to predict compounds that simultaneously modulate amyloid-beta aggregation and neuroinflammatory pathways [119,120,121]. For example, multi-task neural networks have been employed to predict ligands capable of dual inhibition of BACE1 and GSK-3β, both implicated in AD’s progression [121,122]. Moreover, knowledge graph–based embedding models have facilitated the identification of repositioned drugs that act on multiple neurodegeneration-associated targets [123,124], such as donepezil analogs with additional antioxidant or anti-inflammatory properties [125,126].

Antimicrobial resistance (AMR) presents a growing public health crisis, where multi-target therapies offer a way to prevent rapid resistance evolution [23]. The growing threat of AMR has necessitated the development of new therapeutic strategies. Table 4 highlights several important studies where ML has been applied to combat infectious diseases and predict resistance patterns. ML approaches have been used to identify multi-target antibiotics that interfere with essential bacterial processes such as DNA replication, protein synthesis, and cell wall biosynthesis [131,132]. For instance, supervised learning models trained on pathogen-specific compound libraries have predicted antibiotics that simultaneously bind to DNA gyrase and topoisomerase IV—an established strategy to overcome resistance in Gram-negative bacteria [133,134]. In the context of viral infections like COVID-19, network-based ML models have been used to repurpose existing drugs with multitarget activities against both viral proteins and host factors involved in viral entry and replication [135]. These methods offer a scalable means to rapidly identify broad-spectrum antivirals with polypharmacological profiles.

One of the most promising applications of ML in multi-target discovery lies in drug repurposing and personalized therapy [87,156]. Integrative ML frameworks that incorporate patient-specific omics profiles, electronic health records (EHRs), and pharmacogenomic data are now being used to predict individualized drug combinations. For example, ML models have been developed to stratify patients based on molecular subtypes and predict the optimal multi-target regimen that addresses their unique pathway dysregulations [23,157]. In oncology, this approach has led to patient-specific predictions of kinase inhibitor combinations with minimal off-target toxicity [158]. In another instance, reinforcement learning models have been applied to iteratively optimize drug dosing schedules and combinations for rare diseases, using patient-level simulations and prior clinical knowledge [159,160]. This move toward precision polypharmacology is evident in studies that leverage computational methods for drug repurposing and developing personalized strategies. For example, one study applied a DL model based on BERT for text classification to a benchmark dataset of Traditional Chinese Medicine (TCM) clinical records [161]. The research aimed to classify TCM clinical records into five main disease categories, with the BERT-based method achieving state-of-the-art accuracy and F1 scores. In another instance, the PanGPCR system was developed to predict multiple GPCR targets and repurposing potential for GPCR-targeting drugs. This system uses molecular docking to predict binding affinities and targets. PanGPCR can identify a compound’s potential GPCR targets and repurposing potential by generating a ranked list from docking studies, which can be linked to side effect data from the SIDER database [162]. Such personalized systems pharmacology frameworks represent a significant step toward precision polypharmacology, where treatment strategies are tailored not only to disease mechanisms but also to the molecular context of individual patients.

## 5. Challenges and Future Perspectives

Despite the promising potential of ML in multi-target drug discovery, significant challenges remain. A primary obstacle is the lack of high-quality, large-scale datasets, which are often sparse, heterogeneous, and imbalanced, leading to issues like overfitting and poor generalizability [163,164,165]. Specifically, dataset biases are a significant concern, as publicly available databases often overrepresent well-studied compounds and targets. This can result in models that perform well on existing data but fail to generalize to novel chemical spaces or underrepresented target families. Similarly, imbalanced class distributions are a pervasive issue, as drug activity datasets contain a vast majority of inactive compounds. This can cause models to be biased toward the majority negative class, making them insensitive to the rare but crucial active compounds. The problem is further compounded by the challenge of missing data, where incomplete activity profiles or heterogeneous data formats require sophisticated imputation or specialized machine learning methods to build a unified, robust training set. Furthermore, the “black-box” nature of complex models, such as deep learning, hinders interpretability, making it difficult for chemists and regulatory agencies to understand the biological reasoning behind predictions [72,166]. Models also struggle to generalize across different biological contexts and disease states. A final challenge is integrating these computational predictions into experimental and clinical pipelines, as well as addressing ethical and regulatory concerns surrounding data privacy, algorithmic bias, and model reproducibility [73].

The future of multi-target drug discovery will likely be shaped by synergistic advances that address current limitations. Key areas include developing standardized, high-quality datasets and more interpretable ML models to provide clearer biological insights [74]. Advances in transfer learning and domain adaptation will also improve model generalizability across diverse biological contexts [167,168]. The field is moving toward a more collaborative approach, bridging the gap between computational and experimental pipelines. Looking ahead, the emergence of generative AI, federated learning, and the integration of patient-level omics data are expected to accelerate the development of personalized, multi-target therapies, moving us toward a future of precision polypharmacology [169].

## 6. Conclusions

The integration of machine learning into multi-target drug discovery is a transformative shift in pharmacology, moving beyond traditional single-target methods to better address complex diseases like cancer and neurodegeneration. This approach leverages powerful techniques such as deep learning and graph neural networks to enable high-throughput analysis and predictive modeling. While promising applications in cancer therapy and drug repurposing have been demonstrated, significant challenges remain, including data sparsity and a lack of model interpretability. The future of this field depends on advances in data integration, transfer learning, and interdisciplinary collaboration, with generative AI and patient-level omics data poised to further enhance the development of safer, more personalized therapeutic interventions.

## Figures and Tables

**Table 1:** Representative Data Sources for Machine Learning in Multi-Target Drug Discovery.

Database Name	Data Type	Brief Description	URL/Reference
TTD	Therapeutic targets, drugs, diseases	Provides comprehensive information on known and explored therapeutic protein and nucleic acid targets, their associated diseases, pathways, and corresponding drugs.	https://idrblab.org/ttd/ (accessed on 8 August 2025)
KEGG	Genomics, pathways, diseases, drugs	A knowledge base that links genomic information with higher-level functional information, such as biological pathways, diseases, and drug networks.	https://www.genome.jp/kegg/ (accessed on 8 August 2025)
PDB	Protein and nucleic acid 3D structures	A global archive for the experimentally determined 3D structures of biological macromolecules, including proteins and nucleic acids.	https://www.rcsb.org/ (accessed on 8 August 2025)
DrugBank	Drug-target, chemical, pharmacological data	A comprehensive resource that combines detailed drug data with extensive information on drug targets, mechanisms of action, and pathways.	https://go.drugbank.com/ (accessed on 8 August 2025)
ChEMBL	Bioactivity, chemical, genomic data	A manually curated database of bioactive drug-like small molecules, their bioactivities, and associated targets, extracted from medicinal chemistry literature.	https://www.ebi.ac.uk/chembl/ (accessed on 8 August 2025)
BindingDB	Protein-ligand binding affinities	A public database of experimentally measured binding affinities, focusing on interactions between small molecules and proteins.	http://www.bindingdb.org/ (accessed on 8 August 2025)
STITCH	Chemical-protein interactions	A database of known and predicted interactions between chemicals and proteins, integrating data from curated sources, text mining, and experiments.	http://stitch.embl.de/ (accessed on 8 August 2025)
PubChem	Chemical information, bioactivity	A massive repository of chemical compounds and their biological activities, including structures, bioassay results, and patents.	https://pubchem.ncbi.nlm.nih.gov/ (accessed on 8 August 2025)
STRING	Protein–protein interactions (PPI)	A database of known and predicted PPIs. It includes both physical and functional associations derived from experiments and genomic context.	https://string-db.org/ (accessed on 8 August 2025)
PharmGKB	Pharmacogenomic data	A comprehensive knowledge base that curates information on how genetic variations influence an individual’s response to drugs, linking genes, drugs, and diseases.	https://www.pharmgkb.org/ (accessed on 8 August 2025)
ClinicalTrials.gov	Clinical trial information	A public database maintained by the U.S. National Institutes of Health, containing a registry and results of clinical studies from around the world.	https://clinicaltrials.gov/ (accessed on 8 August 2025)
DisGeNET	Gene-disease associations	A discovery platform that integrates information on human gene-disease associations from various data sources and expert curations.	https://www.disgenet.org/ (accessed on 8 August 2025)
Reactome	Biological pathways	An open-source, curated database of biological pathways and molecular processes in humans, providing detailed information on molecular interactions and events.	https://reactome.org/ (accessed on 8 August 2025)
DrugCombDB	Drug combination data	A specialized database for drug combinations, featuring data on synergy, antagonism, and additivity to guide drug combination research.	https://drugcomb.org/ (accessed on 8 August 2025)
NCI-ALMANAC	Drug combination screening data	A large-scale cancer cell line drug combination screening dataset maintained by the National Cancer Institute (NCI).	https://dtp.cancer.gov/ncialmanac/initializePage.do (accessed on 8 August 2025)
O’Neil (Dataset)	Drug combination screening data	A key dataset for drug combination synergy prediction, published by O’Neil et al. in a 2016 study.	[45]

**Table 2:** Representative Studies on Machine Learning Applications for Drug Combination and Synergy Prediction in Oncology.

Data Sources	ML Approaches	Main Research Focus	Key Findings	Ref.
AZ-DREAM Challenges synergy data and an independent validation set from DrugCombDB	A GNN model named SynerGNet, with data augmentation	To develop a GNN model to accurately predict the synergistic effects of drug pairs against cancer cell lines.	SynerGNet achieved a balanced accuracy of 0.68, outperforming traditional ML. Data augmentation further improved accuracy to 0.73, and the model showed strong performance on unseen data from DrugCombDB.	[95]
DrugCombDB and Oncology-Screen datasets	DeepTraSynergy, a multimodal DL approach using transformers and a multitask learning framework	To predict drug combination synergy using a new DL model that integrates multimodal data, including drug-target, protein-protein, and cell-target interactions.	DeepTraSynergy outperformed classical and state-of-the-art models, achieving accuracies of 0.7715 and 0.8052 on the DrugCombDB and Oncology-Screen datasets, respectively. The study confirmed that integrating PPI networks significantly improved prediction.	[96]
DDS data and five independent datasets	DTSyn, a deep neural network (DNN) model using a multi-head attention mechanism	To identify novel drug combinations and understand the mechanisms of drug synergy from a chemical–gene–tissue interaction perspective.	DTSyn achieved the highest area under the curve (AUC) for the receiver operating characteristic curve (ROC) and best true positive rate (TPR) compared to competing methods. The ablation study confirmed the contribution of both transformer encoder blocks, and the model showed improved interpretability by extracting interactions among chemicals and cancer cell lines.	[97]
NCI-ALMANAC dataset	RF, XGBoost	To predict the synergy of unseen cancer drug combinations using a large-scale modeling study to reduce the need for in vitro tests.	The models predicted synergy with high accuracy. The study also found that certain drug classes—alkylating agents, tyrosine kinase inhibitors, and topoisomerase inhibitors—were better predicted, and that restricting predictions to the most reliable ones significantly decreased the error rate.	[98]
NCI-ALMANAC dataset	SYNPRED, an interdisciplinary approach leveraging specifically designed ensembles of AI algorithms	To develop a robust ensemble learning model for predicting anticancer drug synergy while also focusing on data interpretability. The study also aimed to determine the most appropriate synergy metric.	SYNPRED achieved state-of-the-art performance in both classification and regression models, particularly when using the Combination Sensitivity Score. The study also provided insights into which synergy metrics are most effective and achieved data interpretability using feature importance approaches.	[99]
NCI-ALMANAC dataset	SYNDEEP, a DNN-based binary classification model	To develop a novel DNN model for predicting synergistic drug combinations in cancer therapy.	The proposed DNN model achieved high accuracy (92.21%) and AUC (97.32%) in tenfold cross-validation. The integration of different types of physicochemical and genomic features was found to lead to more accurate predictions.	[100]
NCI-ALMANAC dataset	DL methods benchmarking	Systematically evaluate the impact of various methodological choices of multimodal DL models for predicting drug synergy in cancer.	Feature selection based on biological knowledge improved performance. Drug features were more predictive than a baseline model. Molecular fingerprints performed slightly better than learned representations. DL outperformed other ML methods, and an ensemble combining top DL and ML models provided further performance improvements. The models can also learn biologically meaningful associations between drug and cell line features.	[101]
CellMiner database and DrugComb datasets	MARSY, a multitask DL model using two encoders to capture the interplay between drug pairs and cell lines	To develop a computational model to accurately predict cancer drug-pair synergy scores by imputing missing values in sparse datasets.	MARSY outperformed state-of-the-art and traditional ML models. It successfully predicted synergy scores for new cancer drug-pair cell line combinations, and these novel predictions were validated using independent studies.	[102]
O’Neil and NCI-ALMANAC datasets; PubChem and GDSC databases	PermuteDDS, a Permutable feature fusion network	To develop an effective computational method for predicting drug–drug synergy (DDS) in cancer.	PermuteDDS exhibited superior performance on two benchmark datasets and showed good generalization performance on an independent test set. The permutable fusion mechanism was found to be an effective way to combine drug and cell line features.	[103]
DrugComb, Chembl, HURI datasets and others	DGSSynADR, a DL method using a low-rank global attention (LRGA) model and a bilinear predictor	To develop a new DL method to predict synergistic anticancer drug combinations, focusing on improving model generalization and interpretability.	DGSSynADR achieved better performance compared to seven competitive methods. The LRGA model and bilinear predictor were found to be key to improving prediction performance, and a Smooth L1 loss function helped avoid gradient explosion.	[104]
DrugComb, O’Neil, ALMANAC, Oncology Screen, DrugCombDB datasets and others	SynergyX, a multi-modality mutual attention network with a convolution-augmented attention structure	To develop a computational method for interpretable anti-tumor drug synergy prediction by modeling complex biological and drug interactions.	SynergyX showed superior predictive accuracy compared to other models. It also demonstrated multidimensional interpretability, identifying promising drug combinations for potential lung cancer treatment and uncovering drug-gene interactions.	[105]
DrugComb and CCLE databases	JointSyn, a dual-view jointly learning model	To develop a model for personalized drug synergy prediction that is accurate and robust, especially for cross-dataset predictions.	JointSyn outperformed existing models in predictive accuracy and robustness. The dual-view approach captured complementary synergy-related characteristics. The model’s generalization ability was improved with fine-tuning, and it was used to generate a drug synergy atlas for pan-cancer.	[106]
A large-scale oncology screen published by Merck & Co.	DeepSynergy, a DL model using conical layers	To apply DL to predict anticancer drug synergy, using chemical and genomic information.	DeepSynergy significantly outperformed other ML methods, with a 7.2% improvement over the next best method. It achieved a Pearson correlation of 0.73 and a high AUC of 0.90 for classifying novel combinations.	[107]
Multiomics datasets from TCGA and others	AuDNNsynergy, a DL model that uses three autoencoders to encode omics data	To develop a novel DL model for predicting the synergy of pairwise drug combinations in cancer by integrating multiomics data.	AuDNNsynergy outperformed four state-of-the-art approaches, specifically in terms of rank correlation metrics.	[108]
CCLE, GDSC datasets and others	DrugCell, an DL model that integrates tumor genotypes and drug structure to predict response	To develop an interpretable DL model for predicting drug response and designing synergistic drug combinations in cancer.	DrugCell accurately predicts drug responses, stratifies clinical outcomes, and successfully identifies synergistic drug combinations, which were validated through in vitro and in vivo experiments.	[109]
DrugBank, CCLE databases and an independent test set released by AstraZeneca	DeepDDS, a DL model based on a GNN with an attention mechanism	To develop a GNN-based DL model to identify synergistic drug combinations for specific cancer cells.	DeepDDS achieved better performance than both classical and other DL methods on a benchmark dataset. It also outperformed competitive methods by more than 16% in predictive precision on an independent test set. The model’s graph attention network provided interpretability by revealing important chemical substructures.	[110]
AstraZeneca’s drug combination dataset	DREAM challenge, a community-based, competitive benchmarking framework	To evaluate computational strategies for predicting synergistic drug pairs and biomarkers using a large-scale pharmacogenomic screen.	Winning methods achieved an accuracy matching biological replicates for over 60% of combinations. The study also identified a genomic rationale for some synergy predictions, but noted that 20% of combinations were poorly predicted by all methods.	[111]
O’Neil, ALMANAC, CLOUD and FORCINA datasets	MGAE-DC, a multi-channel graph autoencoder (MGAE) model	To develop a MGAE model that can predict the synergistic effects of drug combinations by leveraging not only synergistic data but also additive and antagonistic data.	MGAE-DC consistently outperformed state-of-the-art methods on four benchmark datasets. The model’s approach of integrating non-synergistic data and using an attention mechanism to capture invariant patterns improved its generalization performance and predictive accuracy.	[112]
O’Neil dataset; various online databases and published literature	A GCN model to solve a link prediction task	To develop a GCN model to predict cell line-specific synergistic anticancer drug combinations by integrating multiple biological networks.	The GCN model accurately predicted synergistic drug combinations, with a mean AUC of 0.84 across 39 cell lines. An in-depth literature survey validated many of the top predicted combinations, confirming their synergistic anti-tumor activity.	[113]
DrugComb dataset; cell-line omics database	MatchMaker, a DL framework that uses drug chemical structure and gene expression profiles	To develop a DL model to predict drug synergy scores with high accuracy by using the largest available dataset.	MatchMaker showed significant improvements over state-of-the-art models, with up to ~15% better correlation and ~33% lower mean squared error (MSE). The study also identified drug pairs and cell types that were harder to predict and presented novel candidate pairs.	[114]
DrugCombDB and Oncology-Screen datasets	GraphSynergy, a DL framework using a spatial-based GCN and an attention component	To develop an end-to-end DL framework for predicting synergistic anticancer drug combinations by encoding high-order topological relationships in a PPI network.	GraphSynergy outperformed classic and state-of-the-art models on two datasets, with accuracy values of 0.7553 and 0.7557. The study also found that the model’s attention mechanism highlighted pivotal proteins with relevant molecular functions, improving interpretability.	[115]
Monotherapy data from high-throughput cancer cell line screens, and efficacy data from over 5000 in vitro drug combinations and 26 clinical trials	IDACombo, a method based on the principle of independent drug action (IDA)	To develop a method to predict the clinical efficacy of cancer drug combinations using monotherapy cell line screen data.	IDACombo’s predictions showed high agreement with measured efficacies both in vitro (Pearson’s correlation = 0.93) and in clinical trials (accuracy > 84%). The method provides a framework for prioritizing new combinations.	[116]
Drug combination synergy score dataset; DrugBank and ChEMBL datasets; CCLE and GDSC databases	TranSynergy, a knowledge-enabled DL model using a self-attention transformer	To develop a mechanism-driven, interpretable DNN for synergistic drug combination prediction and pathway deconvolution, with a focus on cancer treatments.	TranSynergy outperformed the state-of-the-art method in performance and interpretability. It revealed novel pathways associated with synergistic combinations and predicted new high-confidence synergistic combinations for ovarian cancer.	[117]

Note: The table provides an overview of key studies, including their data sources, machine learning (ML) approaches, main research focus, and key findings.

**Table 3:** Representative Studies on Machine Learning Applications for Multi-Target Drug Discovery in Neurodegenerative Diseases.

Data Sources	ML Approaches	Main Research Focus	Key Findings	Ref.
DrugBank, DrugCentral, ChEMBL, BindingDB datasets and others	DeepDrug, a GNN model	To propose a novel AI-driven drug repurposing methodology to identify an effective combination of approved drugs for AD.	DeepDrug successfully identified a five-drug lead combination that targets multiple AD-related pathologies, demonstrating a novel expert-guided, AI-driven method for drug combination discovery in neurodegenerative diseases.	[127]
STRING and DrugBank databases	HNNDTA, a hybrid neural network for drug-target affinity (DTA) prediction	To propose a hybrid neural network for DTA prediction to facilitate drug repurposing for AD by identifying potential leads targeting the sigma-1 receptor.	The study identified haloperidol and bromperidol as lead compounds for AD treatment, proposing a new computer-aided drug design approach that is faster and more economical and has the potential for multi-target action.	[128]
BindingDB, ChEMBL, PubChem, and MPD3 datasets	ML virtual screening + molecular dynamics	To identify potential GSK3β inhibitors for treating neurodegenerative diseases, such as AD and PD, using a combined ML and molecular simulation approach.	The study identified three compounds with strong binding scores, with ZINC136900288 showing the highest affinity. These compounds feature novel chemical scaffolds and may serve as promising candidates for future experimental validation as GSK3β inhibitors.	[129]
ChEMBL database; Synapse portal dataset and others	DRIAD, a ML framework that links gene expression to AD pathology	To develop a ML framework for identifying drug repurposing candidates for AD.	The DRIAD method produced a ranked list of potential drug candidates for AD, providing a systematic way to nominate drugs for future clinical trials.	[130]

Note: The table structure and description are consistent with those of Table 2.

**Table 4:** Representative Studies on Machine Learning Applications for Combating Infectious Diseases and Antimicrobial Resistance.

Data Sources	ML Approaches	Main Research Focus	Key Findings	Ref.
CARD, ARDB, and UNIPROT databeses	Two DL models, DeepARG-SS and DeepARG-LS, were developed to predict antibiotic resistance genes (ARG)	To propose a DL approach for accurately predicting antibiotic resistance genes from metagenomic data to address the limitations of traditional bioinformatics methods.	The DeepARG models demonstrated high precision and recall in predicting ARG and consistently outperformed the typical “best hit” approach by yielding lower false negative rates.	[136]
The HMD-ARG-DB, a curated multi-label ARG database	HMD-ARG, an end-to-end Hierarchical Multi-task DL framework for ARG annotation	To develop a multi-task DL framework for the accurate and detailed annotation of ARG from sequence data.	The HMD-ARG method demonstrates superior performance over state-of-the-art methods and can simultaneously identify an ARG’s class, resistance mechanism, and gene mobility.	[137]
ARGNet-DB, a dataset containing 48,615 amino acid sequences	ARGNet, a DNN that combines an unsupervised learning autoencoder with a CNN for classification	To develop a robust, alignment-free DNN to identify and classify a broad range of ARG from sequence data.	ARGNet demonstrated superior performance over other DL models. The model is flexible, efficient, and accurate, making it suitable for both targeted and metagenomic sequencing data.	[138]
DRIAMS database	MSDeepAMR, a DNN model	To propose a DNN model to predict AMR from raw mass spectrometry data.	The MSDeepAMR models showed good classification performance, with AUROC values above 0.83 in most cases. Additionally, adapted models improved AUROC by up to 20% compared to models trained on external data alone.	[139]
NARMS program for genomes and metadata	XGBoost-based models were used to predict minimum inhibitory concentration (MIC) from whole-genome sequences	To develop highly accurate ML models for predicting antimicrobial MIC and identifying associated genomic features in nontyphoidal *Salmonella*.	The models achieved an average accuracy of 95% for predicting MIC. The study also demonstrated that highly accurate models could be generated with a small number of genomes and that the approach is stable over time.	[140]
DRIAMS database	Calibrated classifiers were trained to predict AMR from MALDI-TOF mass spectra profiles	To develop a ML approach to accelerate the determination of AMR directly from clinical MALDI-TOF mass spectra.	The models showed strong predictive potential with AUROC values of 0.80, 0.74, and 0.74 for *S. aureus*, *E. coli*, and *K. pneumoniae*, respectively. A clinical case study showed that this approach would have been beneficial in a majority of cases by providing a faster and more accurate treatment recommendation.	[141]
Microbiome datasets	A ML approach that uses RF to predict and catalog antimicrobial peptides (AMPs)	To discover and catalog novel AMPs from the global microbiome to address the antibiotic-resistance crisis.	The research resulted in the AMPSphere catalog of nearly one million non-redundant peptides. Validation showed that 79 out of 100 tested peptides were active against clinically relevant drug-resistant pathogens.	[142]
Chemical + genomic datasets	Explainable DL using ensembles of GNN and explainable graph algorithms	To use an explainable DL approach to discover a novel structural class of antibiotics to combat antibiotic resistance.	The method successfully identified a new structural class of antibiotics that are selective against drug-resistant pathogens like MRSA and VRE and are effective in mouse models of infection.	[143]
Whole-genome sequence (WGS) data from *Enterobacteriaceae* isolates	A ML algorithm was compared against a rules-based approach for predicting resistance profiles	To evaluate and compare the performance of rules-based and ML approaches for predicting AMR from WGS data.	Both the rules-based and ML approaches achieved high agreement (89.0% and 90.3%, respectively) with standard phenotypic diagnostics. The study also identified specific sources of disagreement for each method.	[144]
Bacterial WGS datasets and phenotypes data	Several ML algorithms, including logistic regression, RF, SVC, and GBTC	To apply ML to WGS data to predict phenotypic polymyxin resistance in *Klebsiella pneumoniae* CG258.	The ML approach with a reference-based genomic data representation outperformed the rules-based approach. The models also correctly identified known resistance genes and suggested potential novel determinants.	[145]
Chemical libraries: Drug Repurposing Hub and ZINC15 database	A DNN model was trained to predict molecules with antibacterial activity	To use DL model to discover new, structurally divergent antibiotics to combat antibiotic resistance.	The DL model successfully identified a molecule, halicin, which is effective against multi-drug resistant pathogens. The approach also identified eight other new antibacterial compounds from a large chemical library.	[146]
Chemogenomics data	INDIGO, a computational approach that predicts synergistic or antagonistic interactions	To develop a computational method to predict effective antibiotic combination therapies that counter drug resistance.	The INDIGO approach significantly outperformed existing methods in predicting antibiotic interactions in *E. coli* and was successfully used to estimate drug-interaction outcomes in other pathogens like *M. tuberculosis* and *S. aureus*.	[147]
Chemogenomic profiles of drugs and metabolic perturbations	MAGENTA, a computational framework that predicts drug–drug interactions	To develop a computational framework to identify robust synergistic antibiotic combinations by predicting their efficacy in diverse microenvironments.	The framework successfully identified synergistic antibiotic combinations that are effective across different environments and accurately predicted changes in drug efficacy.	[148]
Antibiotic combination and PPI network data	A graph learning framework that combines network proximity with network propagation and a graph regularization model	To develop a graph learning framework to predict synergistic antibiotic combinations.	The model showed better performance and interpretability compared to existing methods for predicting synergistic antibiotic combinations.	[149]
Antimalarial compound libraries + in vitro assays	CoSynE: a ML model for predicting antimalarial combination synergy	To use ML to predict novel, synergistic antimalarial drug combinations with a focus on addressing drug resistance.	CoSynE successfully predicted synergistic combinations with significant enrichment over random selection, including with entirely novel compounds.	[150]
DTI and antiviral data, drug–drug combination datasets	A neural network architecture that jointly learns DTI and DDS	To use DL to identify synergistic drug combinations for COVID-19 with limited combination data.	The model performed significantly better than previous methods and successfully predicted two synergistic drug combinations that were validated in vitro.	[151]
AMP and antimicrobial agent synergy datasets	Supervised ML classifiers (RF, SVM) for AMP synergy	To predict the synergistic effects of combining AMP and antimicrobial agents using ML to reduce experimental effort and cost.	The models achieved a high accuracy of 76.92% in predicting synergistic effects. The analysis also revealed the most important features for prediction, which include the target microbial species and the MIC of the agents.	[152]
Large-scale chemical screening databases	MolE, a self-supervised DL framework to learn task-independent molecular representations	To develop a lightweight computational strategy for antimicrobial discovery using pre-trained molecular representations.	The model successfully identified and experimentally confirmed three human-targeted drugs as growth inhibitors of *Staphylococcus aureus* that were structurally distinct from existing antibiotics.	[153]
Broad Institute and other compound databases	DL models were used for virtual screens and toxicity filtering	To discover non-toxic antibiotics that are effective against metabolically dormant bacteria, which are associated with chronic infections and resistance.	The approach successfully identified semapimod, an anti-inflammatory drug that selectively kills stationary-phase bacteria by disrupting the outer membrane.	[154]
Global-scale genomic and metagenomic samples	DRAMMA, a ML method that uses protein properties, genomic context, and evolutionary patterns to detect novel genes	To develop a ML method for detecting novel ARGs in metagenomic data.	DRAMMA demonstrated robust predictive performance and holds promise for the discovery of novel ARGs that lack sequence similarity to any known resistance genes.	[155]

Note: The table structure and description are consistent with those of Table 2.

## Data Availability

Not applicable.

## Abbreviations

PK: pharmacokinetics; PD: pharmacodynamics; DTI: drug-target interactions; ML: machine learning; SVMs: support vector machines; RFs: random forests; DL: deep learning; GNNs: graph neural networks; GCNs: graph convolutional networks; GATs: graph attention networks; MLP: multi-layer perceptron; PPI: protein–protein interaction; NCI: National Cancer Institute; k-NN: k-nearest neighbors; NB: naïve Bayes; MTL: multi-task learning; RNNs: recurrent neural networks; AE: autoencoder; VAE: variational autoencoder; AI: artificial intelligence; GANs: generative adversarial networks; GM: gut microbiota; LoSM: locked-state model; DNN: deep neural network; AUC: area under the curve; ROC: receiver operating characteristic curve; TPR: true positive rate; DDS: drug–drug synergy; LRGA: low-rank global attention; MGAE: multi-channel graph autoencoder; MSE: mean squared error; IDA: independent drug action; AD: Alzheimer’s disease; PD: Parkinson’s disease; DTA: drug-target affinity; AMR: antimicrobial resistance; ARG: antibiotic resistance genes; MIC: minimum inhibitory concentration; AMPs: antimicrobial peptides; WGS: whole-genome sequence; EHRs: electronic health records; TCM: Traditional Chinese Medicine.
